# Treatment of large persistent tracheoesophageal peristomal fistulas using silicon rings^☆^

**DOI:** 10.1016/j.bjorl.2016.06.011

**Published:** 2016-07-21

**Authors:** Ibrahim Erdim, Ali Ahmet Sirin, Bahadir Baykal, Fatih Oghan, Ali Guvey, Fatma Tulin Kayhan

**Affiliations:** a Bakirkoy Sadi Konuk Education and Research Hospital, Department of ORL, Istanbul, Turkey; b Dumlupinar University, Faculty of Medicine, Department of ORL, Kutahya, Turkey

**Keywords:** Voice prosthesis, Silicon ring, Fistula, Prótese vocal, Anel de silicone, Fistula

## Abstract

**Introduction:**

Tracheoesophageal peristomal fistulae can often be solved by reducing the size of the fistula or replacing the prosthesis; however, even with conservative techniques, leakage around the fistula may continue in total laryngectomy patients. Also, several techniques have been developed to overcome this problem, including injections around the fistula, fistula closure with local flaps, myofascial flaps, or free flaps and fistula closure using a septal perforation silicon button.

**Objective:**

To present the results of the application of silicon ring expanding the voice prosthesis in patients with large and persistent peri-prosthetic fistula.

**Methods:**

A voice prosthesis was fitted to 42 patients after total laryngectomy. Leakage was detected around the prosthesis in 18 of these 42 patients. Four patients demonstrated improvement with conservative methods. Eight of 18 patients who couldn’t be cured with conservative methods were treated by using primary suture closure and 4 patients were treated with local flaps. As silicon ring was applied as a primary treatment in the 2 remaining patients and also, applied to 2 patients who had recurrence after suture repair and to 2 patients who had recurrence after local flap implementation. Silicon rings were used in a total of 6 patients due to the secondary trachea-esophageal fistula. Patients were treated with provox-1 initially and later with provox-2. At the time of leakage around the fistula, 6 patients had provox-2.

**Results:**

Fistulae were treated successfully in 6 patients, and effective speech of patients was preserved. Patients experienced no adaptation problem. Prosthesis changing time was not different between silicon rings expanded and normal prosthesis applied patients. Silicon ring combined voice prosthesis was used 26 times; there was no recurrence in fistula complication during 29 ± 6 months follow up.

**Conclusion:**

Silicon rings for modified expanded voice prosthesis seems to be an effective treatment for persistent peri-prosthetic leakage, for both, fistula closure and preserving the patients speech.

## Resumo

**Introdução:**

Fístulas traqueoesofágicas persistentes podem ser resolvidas através da redução do tamanho da fístula ou substituição da prótese; no entanto, mesmo com técnicas conservadoras, o pertuito em torno da fístula pode continuar em pacientes com laringectomia total. Além disso, várias técnicas têm sido desenvolvidas para superar esse problema, incluindo injeções ao redor da fístula, fechamento da fístula com retalhos locais, retalho miofasciais, ou retalhos livres e fechamento da fístula usando um botão septal de silicone.

**Objetivo:**

Apresentar os resultados da aplicação de anel de silicone para expansão da prótese vocal em pacientes com grandes fístulas periprotéticas persistentes.

**Método:**

Prótese vocal foi colocada em 42 pacientes após laringectomia total, e fístula foi detectada ao redor da prótese em 18 desses 42 pacientes. Quatro pacientes obtiveram melhora com métodos conservadores. Oito dos 18 pacientes que não obtiveram sucesso com métodos conservadores foram tratados usando sutura primária e quatro pacientes foram tratados com retalhos locais. Um anel de silicone foi aplicado inicialmente nos dois pacientes restantes e, também, aplicado a dois pacientes que tiveram recorrência após a técnica de sutura e a dois pacientes que tiveram recorrência após a utilização de retalho local. No total, seis pacientes receberam anéis de silicone em decorrência da fístula traqueoesofágica secundária. Os pacientes haviam sido tratados com provox-1 inicialmente e posteriormente com provox-2. No momento detecção da fístula em torno do estoma, seis pacientes haviam recebido provox-2.

**Resultados:**

A fístula foi tratada com sucesso em seis pacientes. Além disso, após o tratamento a fala foi mantida de forma eficaz nos pacientes. Não houve problema de adaptação. O tempo de troca da prótese expandida com os anéis de silicone não foi diferente do tempo que se leva para a colocação da prótese normal. O anel de silicone combinado com a prótese vocal foi usado 26 vezes em pacientes na época da troca de prótese e não houve recorrência da fístula durante os 29 ± 6 meses de acompanhamento.

**Conclusão:**

Os resultados sugerem que em casos de grandes fístulas peri-prostéticas persistentes, anéis expandidos de silicone e prótese vocal modificada, são eficazes tanto para o fechamento da fístula como para a manutenção da fala do paciente.

## Introduction

One of the most important problems of patients who have undergone total laryngectomy is loss of speech. A voice prosthesis can solve the problem in most patients; however, there can be numerous complications, including a peristomal fistula.^1^, ^2^, ^3^, ^4^ This frequent complication can result in severe morbidity, including aspiration pneumonia and malnutrition, or even mortality.^5^, ^6^, ^7^

Such problems can be solved by reducing the size of the fistula or replacing the prosthesis; however, even with conservative techniques, leakage around the fistula may continue. Several techniques have been developed to overcome this problem, including injections around the fistula^8^, ^9^, ^10^, ^11^; fistula closure with local flaps,^12^ myofascial flaps,^1^, ^13^, ^14^ free flaps;^1^, ^5^ and fistula closure using a septal button.^6^, ^13^

The problems caused by small fistulas (5–10 mm) are easier to overcome compared to those caused by large fistulas; indeed, it may not be possible to solve the problems caused by large fistulas, and complications such as speech loss and morbidity may result from intervention.

Here, we report the application of a silicone ring expanded voice prosthesis in patients who had a large-sized fistula and persistent peri-prosthetic leakage.

## Methods

A voice prosthesis was fitted to 42 patients after total laryngectomy between January 2005 and December 2011. Ethical approval was obtained from ethical committee as a number of 32/2015. Leakage was detected around the prosthesis in 18 of the 42 patients. While four patients improved with conservative methods, eight patients improved at first with conservative methods but later did not respond to therapy, and six patients did not respond to conservative therapy even initially.

Eight of fourteen patients who could not be cured with conservative methods were treated using primary suture repair, and four patients were treated with local flaps. A silicone ring was applied initially to two patients with wide fistulas. A silicone ring was also applied to two patients who had recurrence after suture repair and two patients who had recurrence after local flap implementation. In total, silicone rings were fitted to six patients.

Five of the patients who received Provox 2 widened with silicone rings were male and one was female. The mean age was 57 ± 11 years. Four patients underwent bilateral functional neck dissection, one underwent bilateral functional neck dissection and right submandibular gland excision, and one underwent right functional, left radical neck dissection and reconstruction with a pectoralis major myocutaneous flap. Three patients received radiotherapy, one patient received radiotherapy and chemotherapy, and two patients received neither. A secondary tracheoesophageal fistula was opened in all patients. The patients were treated with Provox 1 first and followed later with Provox 2. At the time of leakage around the fistula, six patients had Provox 2 (outer diameter, 22.5 F; Atos Medical, Hörby, Sweden). The minimum and maximum diameters of the fistulas were 1.5 cm × 1.5 cm and 2 cm × 2.5 cm, respectively.

### Preparation and application of the silicone ring expanded voice prosthesis

**Figure 1:**
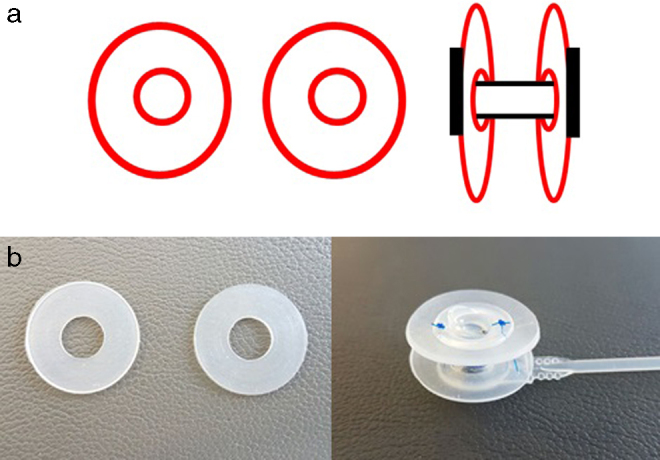
(a) Schematization of expanded voice prosthesis with silicone rings. (b) Silicone rings and prepared expanded voice prosthesis.

**Figure 2:**
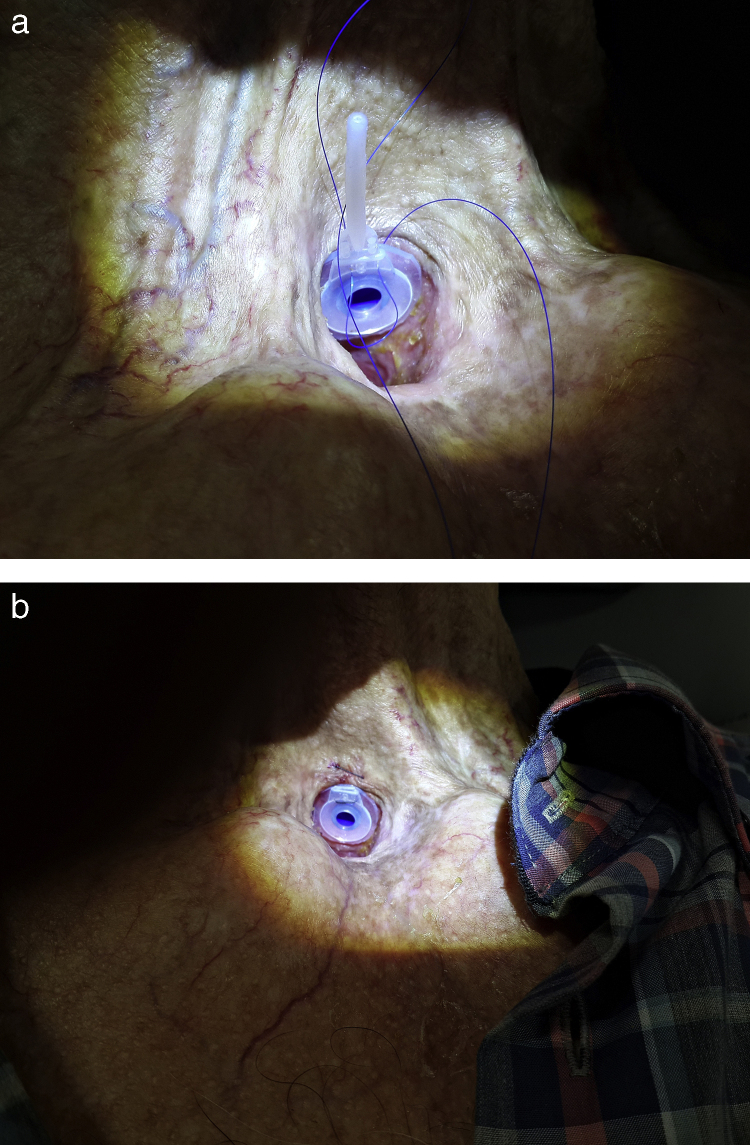
(a) The rings engaged with the tracheal and esophageal flanges of the voice prosthesis. (b) He upper side of the tracheal flange of the voice prosthesis was sutured to skin on the tracheostoma using 3.0 non-absorbable sutures.

Two silicone rings made of wings of septal buttons or inexpensive silicone plaques were prepared according to the width of the fistula and inner diameter of the voice prosthesis. These rings engaged with the tracheal and esophageal flanges of the voice prosthesis (Fig. 1a). The silicone rings were fixed to the voice prosthesis using 3.0 non-absorbable sutures – 2 at the posterior flange and 2 at the anterior flange (Fig. 1b). Care was taken that the prepared rings did not have sharp pieces on the outer side. The wing matched with the esophagus engaged with the fistula. The voice prosthesis combined with the silicone rings was applied to the tracheoesophageal fistula. Last, the upper side of the tracheal flange of the voice prosthesis was sutured to skin on the tracheostoma using 3.0 non-absorbable sutures (Fig. 2a and b).

## Results

Fistulas were treated successfully in six patients. After treatment, effective speech of the patients was maintained. No adaptation problem occurred in the patients. A silicone ring combined with a voice prosthesis was used 26 times in patients when it was time to change the prosthesis, and there was no recurrence of fistula complications during 29 ± 6 months of follow-up. Prosthesis changing time was 183.5 ± 58.7 (min–max: 21–424) days for total 42 patients, and 171.6 ± 74.8 (min–max: 32–384) days for silicon ring expanded prosthesis applied patients. Prosthesis changing time was not statistically different between these two groups (Non-parametric Mann–Whitney *U* test was used and *p* = 0.163).

## Discussion

The size of the fistula and whether the patient has received radiotherapy are essential factors in the closure of tracheoesophageal fistulas. The success rate of fistula closure is decreased in patients who received radiotherapy, especially when surgical techniques were applied.^1^, ^14^ However, septal button^6^, ^13^, ^15^ and silicone ring expanded Provox, which we used, are more effective in patients who have received radiotherapy because these techniques have no effect on wound healing.

In most patients, the problem can be solved by using a smaller prosthesis. If the problem persists, spontaneous closure of the fistula can occur after detaching the prosthesis. However, if fistula closure with this method fails, the application of various techniques could be required according to the size of the fistula.^16^

For small fistulas (5–10 mm), local suture-repair techniques are appropriate and the success rate is between 60% and 100%.^14^ Additionally, some studies have reported the effectiveness of collagen,^8^ hyaluronate,^9^ calcium hydroxyapatite,^10^ and GM-CSF^11^ injections for small fistulas.

Jacobs et al.^17^ used the “submucosal purse-string suture” technique in 20 patients to constrict the fistula and reuse the voice prosthesis. They were successful in 16 of 20 patients (80%). While they achieved success in 9 of 16 patients in the first suture trial, repetitive suturing was required for the remaining 7 patients. This technique is simple and could be the first choice method for constricting fistulas. However, it cannot be considered effective for medium- or large-sized fistulas. Additionally, tracheostoma narrowness, which excluded one patient from the study, makes the application of this technique difficult.^17^ Lee et al.^12^ reached the fistula tract by making an incision from the 9 to 3 o’clock direction on the upper part of the tracheostoma. They elongated the incision toward the sternocleidomastoid (SCM) muscle. After splitting the trachea and esophagus, they repaired the esophageal defect with absorbable sutures. Rotating the inferiorly-based flap, using the right SCM muscle, they sutured this on the repaired esophageal site. Also, they repaired the trachea using absorbable sutures. The patient's fistula was closed completely, and the patient could not speak with the voice prosthesis. The limitations of this study are that the diameter of the fistula was not stated and the technique was tried on only one patient.^12^ Additionally, Wong et al.^15^ used an SCM muscle flap twice consecutively to close a tracheoesophageal fistula, but they were not successful. Therefore, they closed the fistula with a septal button.

Mobashir et al.^16^ removed the fistula, making an incision from the 9 to 3 o’clock direction on the upper part of the tracheostoma in middle-sized fistulas (maximum fistula size, 1.5 cm × 1 cm). They put non-absorbable sutures on the tracheal and esophageal parts of the fistula and closed the fistula by tying. In all patients, the fistula closed successfully, but the patients could not speak.

For large fistulas, large-based flaps and free flaps could be used. The pectoral major myofascial flap (PMMF)^13^ and radial forearm free flap are major types of flaps used for large fistulas. In these surgeries, there can be morbidity at the donor sites. Radial flaps are more suitable than PMMFs because they are thin and shaped easily. With a PMMF, dysphagia and constriction of the tracheal lumen may occur because of the mass effect. With a radial forearm free flap, microvascular anastomosis is needed. This is technically difficult, and the operation time is long. Also, the vascular status of the patient is important.^14^ Despite this surgical technique, the closure of large-sized fistulas could not be achieved in previous reports.^5^, ^13^ In particular, in cases where the vessel in the pedicle could not nourish the flap, closure of the fistula could not be achieved because of necrosis.

An alternative approach for a large-sized fistula is a septal button.^6^, ^13^, ^15^ Septal buttons can be applied easily and rapidly and are well tolerated. They prevent aspiration and pulmonary infections, and patients can eat and drink comfortably. Salivary bypass tubes (Boston Medical Products, Westborough, MA) can also be used for eating and drinking in patients with large fistulas. However, depending on the duration of usage of the tube, large granulomas may develop on the tip and feeding can deteriorate,^13^ making the patient uncomfortable. Another uncomfortable situation is the requirement for suture repair of the salivary bypass tube to neck skin to stabilize it.^13^ For these reasons, septal button usage is recommended for large fistulas, or for failure of the flaps used for fistula closure.^6^, ^13^, ^15^ When avoidance of surgical morbidity is required and if there are medical contraindications, this technique could be recommended.^13^ However, the disadvantages of a septal button are that the patient cannot speak again, and there can be fungal proliferation around the button.^15^

Hilger et al.^18^ demonstrated the treatment of tracheoesophageal fistulas by attaching a silicone ring to the tracheal flange of the prosthesis. They prevented fistula recurrence in 29 of 32 patients but failed in 3 patients; subsequently, in 9 patients, the fistula relapsed and additional interventions were needed. Therefore, in 20 of 32 patients they obtained successful results, but in 12 patients (37.5%) the fistula could not be treated using this method alone. In this study, for either small or large fistulas, the same technique was attempted on all patients. In our study, 2 patients with a local flap who had a relapse, 2 patients who had a relapse after suture repair, and 2 patients who had very large fistulas (6 patients in total) received intervention with silicone rings. The difference between our technique and that of Hilger et al.^18^ is the attachment of the silicone ring to both the tracheal and esophageal flanges of the voice prosthesis instead of attaching the silicone ring to only one side. In this way, we aimed to prevent recurrent aspiration.

Eric Blom designed adjustable a bi-flanged fistula prosthesis (Blom-Singer^®^) made from medical-grade silicone for the management of hypopharyngeal fistulas. Our silicone rings are similar to this prosthesis but our rings are used to prevent tracheoesophageal fistulas. The prosthesis flanges designed by Blom are soft, flexible, and translucent like our rings.

With a septal button, the patient cannot speak again despite closure of the tracheoesophageal fistula. Similarly, when the fistula is closed with flaps, the patient cannot speak. Also, there is a failure risk with flaps because of necrosis of the flap or opening of the sutures on the flaps. However, using our technique, the fistula was closed and the patient retained the ability to speak. Additionally, the need for surgery was prevented. In patients who have received radiotherapy, the success rate of fistula closure decreases^1^, ^14^ with either local or other surgical techniques. In our study, the success rate was independent of radiotherapy. However, one of the disadvantages of our technique is the need to prepare silicone rings every time the prosthesis is changed.

## Conclusions

Although we demonstrated our technique only in six patients, we managed to treat resistant enlarged tracheoesophageal fistula while preserving speech without periprosthetic leakage recurrence. According to our study the use of expanded voice prosthesis with silicone rings for large tracheoesophageal fistulas with persistent leakage appears to be effective.

## Conflicts of interest

The authors declare no conflicts of interest.
